# Odontogenic Cutaneous Fistula: A Cause of Persistent Cervical Discharge

**DOI:** 10.1155/2018/3710857

**Published:** 2018-06-11

**Authors:** Nicholas Figaro, Solaiman Juman

**Affiliations:** Department of Clinical Surgical Sciences, University of the West Indies, Eric Williams Medical Sciences Complex, Champs Fleur, Trinidad and Tobago

## Abstract

Odontogenic cutaneous fistulas often lead to intense levels of patient discomfort and suffering. Due to its rarity and the absence of dental symptoms, a considerable number of patients are usually misdiagnosed which results in inappropriate management. This case report presents a 16-year-old patient with a 2-year history of a nonhealing, persistently discharging lesion in the left submandibular region of the neck. The patient underwent exploration of the left submandibular region, and a fistulous tract directed superomedially to the ipsilateral lower molar teeth was excised. A subsequent panoramic orthopantomogram performed one week postoperatively demonstrated radiolucency is the distal root of tooth 37. A final diagnosis of odontogenic cutaneous fistula was made, and the patient was referred to the Maxillofacial Department for treatment of the offending tooth.

## 1. Introduction

An odontogenic cutaneous fistula is one of the many consequences of chronic dental infection and is defined as an abnormal communication between the face and the oral cavity [1]. A breach in the enamel and the dentine by a carious lesion, trauma, or another cause provide a portal for bacterial invasion. Failure to treat the affected tooth during the early inflammatory stage sets the precedent for further complications. Typically, the absence of prompt diagnosis and treatment results in pulp necrosis and the spread of infection beyond the affected tooth into the periradicular tissues, which often lead to an apical periodontitis. The chronic process slowly evolves through the cancellous alveolar bone, following the path of least resistance until it perforates the cortical plate of the mandible, spreading to the surrounding soft tissues and erupting on the skin [2, 3].

Misdiagnosis and inappropriate treatment are common for odontogenic cutaneous fistulas due to its rarity and lack of dental symptoms [4, 5]. The expansive differential diagnosis such as epidermal cysts, furuncle, carbuncle, branchial cleft fistula, pyogenic granuloma, salivary gland fistula, actinomycosis, thyroglossal tract fistula, basal cell and squamous cell carcinoma, osteomyelitis, and foreign body reaction further compound the diagnostic dilemma [5]. The cutaneous fistulous tracts of the face are more frequently caused by dental lesions than any other pathological condition [6]. The variability of clinical morphological presentations and locations of the cutaneous lesions coupled with the lack of knowledge that such a condition can have a dental etiology generally leads to the misdiagnoses by surgeons and dermatologists, leading to unnecessary antibiotic and surgical therapies [1, 6–8]. This is a case report of a 16-year-old female patient with an odontogenic cutaneous fistula which was successfully treated with surgical fistulectomy in the Otolaryngology Department at Eric Williams Medical Sciences Complex (EWMSC), Trinidad and Tobago.

## 2. Case Report

A healthy 16-year-old student was referred to the Department of Otolaryngology with chief complaints of a periodical nonpurulent bloody discharge from a tiny nonhealing aperture located below the body of the left mandible. The patient indicated that 2 years previously she observed a left submandibular swelling without any antecedent coryzal symptoms, fever, pain, or discomfort. The patient also disclosed that she had seen many doctors and was prescribed several courses of antibiotics and had undergone surgery to remove the lesion. Because of persistence of the discharge, which was significantly affecting the quality of her life, she was referred to the ENT Department at the EWMSC.

**Figure 1:**
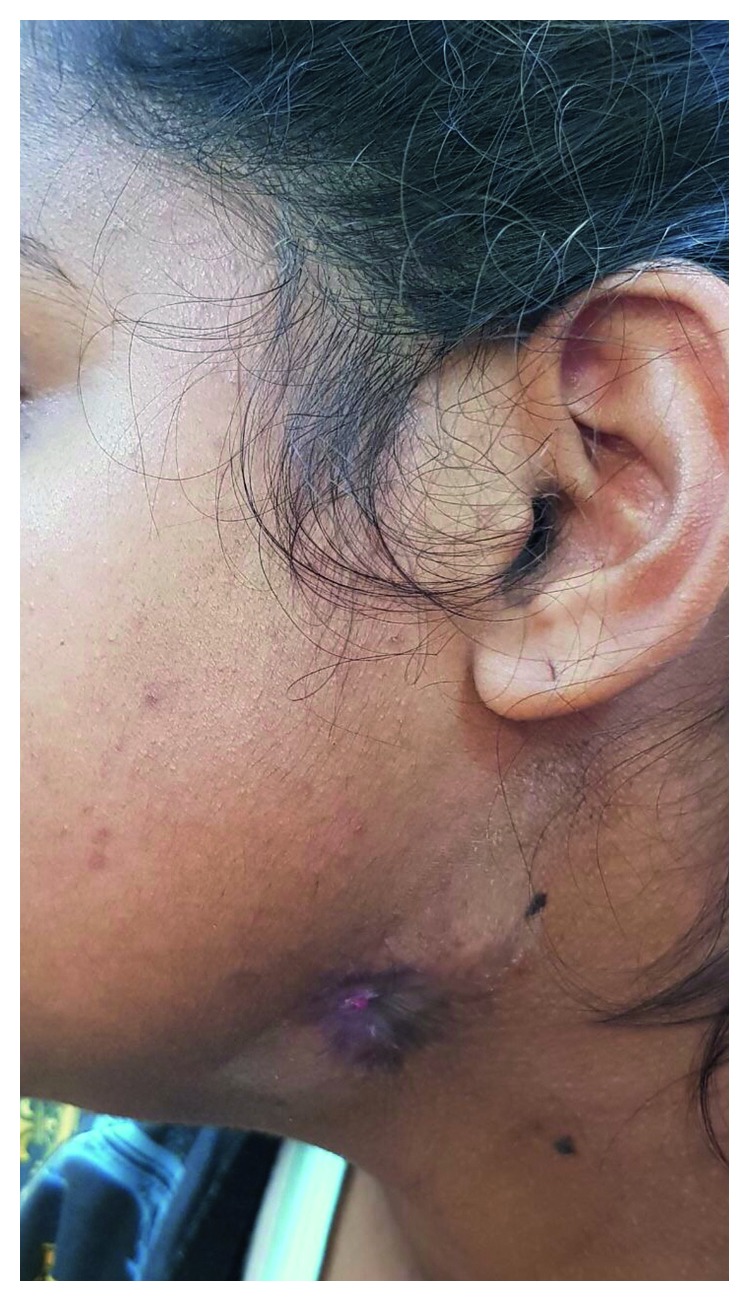
Odontogenic cutaneous fistula with hyperpigmented retracted skin.

Physical examination revealed a nontender 1 × 1 cm hyperpigmented retracted lesion with a 3 mm center of granulation tissue (Figure 1). Gentle pressure of the surrounding skin expressed scanty nonpurulent discharge. Intraoral examination was unremarkable. The working diagnosis at this point in time was a congenital fistula for which an MRI was ordered. The MRI scan discovered a 1.2 × 0.8 × 1.4 cm high T2/STIR low T1 signal intensity area in the left submandibular region along a 0.8 cm tract superomedially. No direct connection to any epithelialized structure was identified. Following extensive discussion with the patient and relatives, the decision was made to perform surgical exploration and surgical fistulectomy.

**Figure 2:**
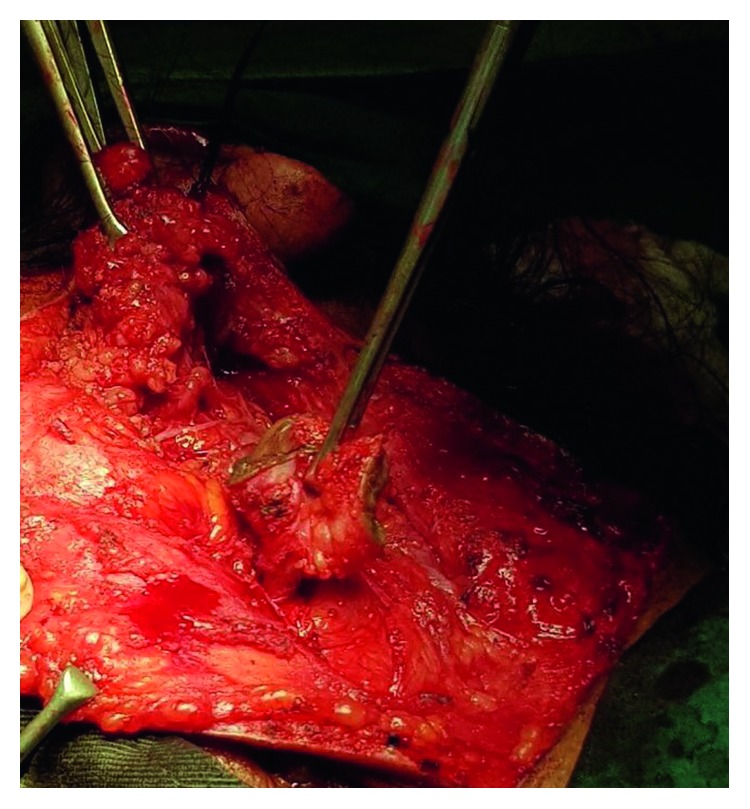
Intraoperative fistula tract dissection.

**Figure 3:**
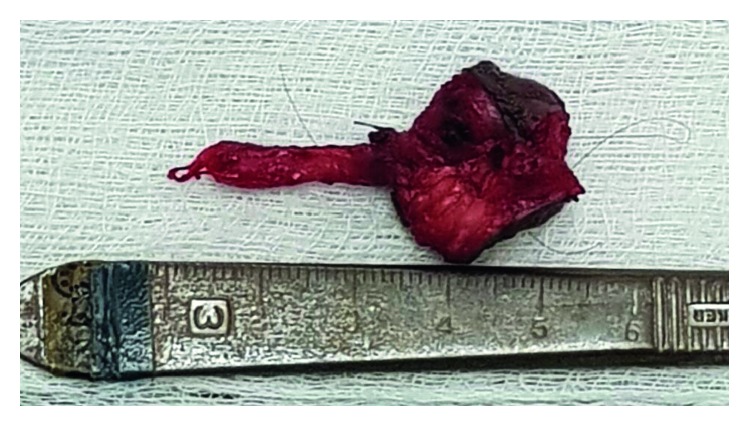
Resected fistula tract.

A primary consideration in the surgery was the protection of the lower branches of the facial nerve in the light of the scarring caused by the recurrent infections and previous surgery. Using the Modified Blair incision, a left superficial parotidectomy was performed with identification and preservation of the lower branches of the facial nerve (Figure 2). A circumferential skin incision around the fistulous opening was deepened, and then the tract was dissected from the surrounding tissue (Figure 3). The fistulous tract coursed superior medially and terminated on a cavity in lower medial border of the left mandible.

**Figure 4:**
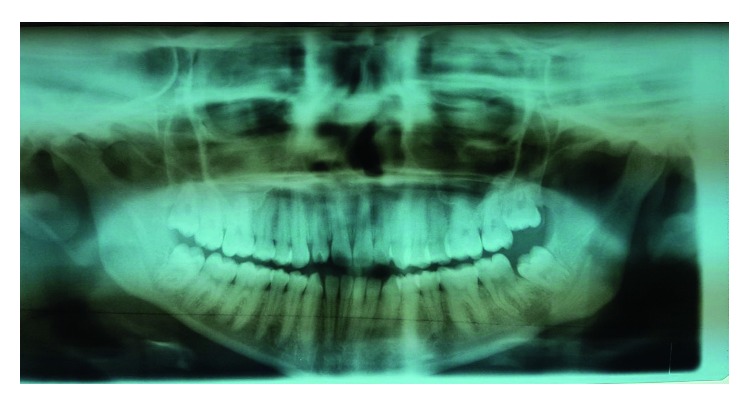
Panoramic radiograph. Radiolucent lesion of the periapical area of the mandibular molar (#37).

Postoperatively, the patient had a transient left marginal mandibular nerve weakness which quickly resolved. Based on the intraoperative findings, an orthopantomogram (OPG) was requested postoperatively. The OPG demonstrated a fully dentate patient with a moderately defined radiolucency extending mesiodistally from the mesial root of #37 to the mesial aspect of #38 and also inferiorly to the lower border of the mandible (Figure 4). Further intraoral periapical imaging confirmed the presence of the osteolytic lesion and a knife edge root resorption of the distal root of #37. Histopathological analysis of the specimen depicted lobules of mature adipocytes, fibrocollagenous tissue with moderate mixed inflammatory infiltrate, and areas of hemorrhage. Based on the history, and the intraoperative and radiological findings, the diagnosis of an odontogenic cutaneous fistula was made and the patient was sent for further care at the Department of Oral and Maxillofacial Surgery.

## 3. Discussion

In the case presented, the clinical diagnosis of an odontogenic cutaneous fistula was achieved postoperatively. The diagnosis was elusive in the absence of carious teeth and an unremarkable dental history, a clinical phenomenon reported in 50% of patients [4, 8–10]. The diagnostic conundrum was compounded by the location of the dental infection to the fistulous aperture and its variability [1, 3, 9]. The nonspecific skin manifestation of an odontogenic cutaneous fistula may mimic a number of other disorders previously mentioned, justifying yet another reason why many patients seek care from a multitude of physicians before an accurate diagnosis is achieved [1, 3, 7, 11]. Our case demonstrates that failure of medical practitioners to effectively communicate and the lack of knowledge of odontogenic cutaneous fistulas lead to diagnostic and therapeutic misadventures as seen in this patient.

A high degree of clinical suspicion is required for an early and correct diagnosis in the absence of an odontogenic etiology [1, 2]. Comprehensive history taking and intraoral examination complete with pulp viability testing and dental X-rays are usually sufficient for a diagnosis [9]. Cone beam CT is unmatched when plane radiography is equivocal or inefficient in determining the extent and the relations of the periapical lesion with the adjacent teeth [2, 8]. Fistulography via X-ray or CT using a gutta-percha usually provides the diagnosis when all other modalities fail [2, 4]. Several authors describe treatment as removing the original source of infection by means of endodontic or extraction therapy with spontaneous closure of the fistulous tract within as much as 2 weeks [1, 2, 8, 10]. Notably, surgical revision or fistulectomy is usually reserved for those cases whose fistulous tracts fail to terminate and those patients with aesthetically unpleasant hyperpigmented scared tissue [3, 4, 9]. In this case, the fistula's aperture seemed to be in close proximity to the distribution of marginal mandibular branch of the facial nerve. As such, a preliminary superficial parotidectomy with wide exposure was adopted to aid in identification and preservation of the branches of the facial nerve. Once the facial nerve branches were identified, an elliptical incision was made around the fistulas opening and the fistulous tract meticulously stripped.

## 4. Conclusion

Our case emphasizes that dental etiology should be considered for a persistent cutaneous fistula of the cervicofacial region. To achieve proper diagnosis of such a rare phenomenon, communication and a consensus among the medical specialists is crucial. Once dental etiology is suspected, a thorough dental examination and dental radiography usually confirms the diagnosis. Dental extraction or endodontic treatment is usually sufficient to eliminate the infection and, therefore, resolve the cutaneous discharge. Surgical intervention is mostly reserved for persistent fistulas and unaesthetically pleasing lesions.
